# The depressed frail phenotype as a risk factor for mortality in older adults: A prospective cohort in Peru

**DOI:** 10.1016/j.heliyon.2021.e08640

**Published:** 2021-12-20

**Authors:** Gabriel A.J. Vasquez-Goñi, Basilio M. Papuico-Romero, Diego Urrunaga-Pastor, Fernando M. Runzer-Colmenares, José F. Parodi

**Affiliations:** a Universidad Científica del Sur, Facultad de Ciencias de la Salud, Carrera de Medicina Humana, Lima, Perú; b Universidad de San Martin de Porres, Facultad de Medicina Humana, Centro de Investigación del Envejecimiento (CIEN), Lima, Perú

**Keywords:** Frailty, Depression, Older adults, Mortality, Latin America, Peru

## Abstract

**Introduction:**

Frailty and depression can coexist as depressed frail phenotype, useful for the comprehensive evaluation of older adults and prevention of adverse outcomes. The objective of this study was to evaluate the role of the depressed frail phenotype and its components as risk factors for mortality in older adults of the *Centro Médico Naval* (CEMENA) of Peru during 2010–2015.

**Material and methods:**

We carried out a secondary data analysis of a prospective cohort that included older adults (60 years and older) treated in the Geriatrics service of CEMENA between the years 2010–2015. Frailty was defined as the presence of three or more Fried phenotype criteria and depression was determined using a Yesavage ultrashort scale score of three or more. The presence of both conditions was defined as depressed frail phenotype. In addition, sociodemographic characteristics, medical and personal history, and performance-based measures were included. We employed crude and adjusted Cox regression models to evaluate the association of interest and estimate Hazard Ratios (HR) with their respective 95% confidence intervals (95% CI).

**Results:**

946 older adults were included in the analysis, with a mean age of 78.0 ± 8.5 years. 559 (59.1%) were male, 148 (15.6%) were found to be frail, 231 (24.4%) had depressive symptoms, 105 (11.1%) had depressed frail phenotype, and 79 (8.3%) participants died during follow-up. The adjusted Cox regression analysis revealed that depressed frail phenotype (HR = 3.53; 95%CI: 2.07–6.00; p < 0.001) was a risk factor for mortality in older adults.

**Conclusions:**

The depressed frail phenotype was associated with a higher risk of mortality in older adults. It is necessary to develop longitudinal studies that allow estimating this phenotype's impact on mortality and evaluate interventions to improve quality of life and reduce the risk of adverse outcomes.

## 1 Introduction

Frailty is a condition defined by the presence of weakness, sedentary behavior, slow gait speed, exhaustion, and unintentional weight loss [1]. This syndrome is associated with a state of vulnerability that increases the risk of falls, disability, hospitalizations, morbidity and death [2, 3]. There are various definitions of frailty, but the most widely accepted is the frailty phenotype, which is the sum of conditions that lead to a dependent and vulnerable state, and the accumulation of deficit based on a pre-established disease that consumes physiological reserves and places older adults in a vulnerable state [4]. Nonetheless, according to the definition of frailty chosen, the prevalence may vary, being described as 13% worldwide and 21.7% in South America [5, 6]. Therefore, frailty represents a public health priority given the increase in the number of older adults in the latter region, and it is not the only relevant geriatric syndrome in South America.

Depression in older adults represents another relevant condition which has a prevalence ranging from 2.8% to 11.8% [7, 8, 9]. It is of note that the management of depression in older adults is challenging. This is due to possible underestimation of its presence in this population and the difficulties for treatment considering the high frequency of comorbidities and cognitive impairment [10]. Depression has also been associated with an increased risk of disability, hospitalizations, cognitive impairment, frailty and mortality, and thus, it must be diagnosed and treated in a timely manner [11, 12, 13]. Along this line, depression and frailty are two associated conditions, in which a deficit of dopaminergic neurotransmitters affects physiological reserves, generating cognitive and motor impairment in older adults [14, 15].

Depression and frailty can present in different ways: they can coexist with an illness, be circumscribed in the clinical picture of a major illness, or they can occur bi- or unidirectionally [13, 16]. A systematic review evaluated the bidirectionality and unidirectionality between depression and frailty and revealed that an older adult with depression had three times the risk of frailty, while an older adult with frailty had approximately two times the risk of presenting depression [17] reinforcing the idea of unidirectionality between depression and frailty. However, it is more relevant to explore the impact that both conditions have on mortality of older adults.

Frailty and depression have been evaluated as predictors of disability [4], hospitalizations [4], and mortality [18, 19, 20] in older adults. Although both conditions can coexist as a syndrome called the depressed frail phenotype [14, 15], frailty follows a more orderly pathway to predict adverse outcomes; however, depression can also be affected by other comorbidities in older adults. Both conditions together can serve for the comprehensive evaluation of older adults in relation to adverse outcomes such as mortality [14, 15, 16, 20, 21]. Previous studies on this topic [15, 20, 22] reported non-conclusive results, and therefore, the objective of this study was to evaluate the role of the depressed frail phenotype and its components as predictors of mortality in older adults at the *Centro Médico Naval* of Peru from 2010-2015.

## 2 Materials and methods

### 2.1 Study design, population and sample

This study is a secondary analysis of a prospective cohort including 1891 older adults (60 years and over) from the Geriatrics Service of the *Centro Médico Naval* (CEMENA) "Cirujano Mayor Santiago Távara” recruited during the period from 2010-2015. The primary study evaluated the prevalence and factors associated with frailty in the study sample. The database is open access and can be downloaded from Figshare [23]. Participants with severe cognitive impairment, defined by a Mini-Mental State Examination (MMSE) score less than 17, were excluded [24]. Likewise, we excluded participants with a positive result in the clock test and those who did not have the variables of interest or did not agree to participate of the study.

Participants were enrolled annually until 2015, adding a new group of patients chosen by non-probability sampling each year until 2015. We did not perform an additional measurement to the baseline assessment in the participants, only mortality was assessed during follow-up. Of the 1,891 participants recruited, 577 were excluded for having an MMSE score less than 17 or having a positive result on the clock test and 368 for not having complete information for the variables of interest. Finally, we analyzed 946 participants. A statistical power of 100% was obtained, considering 946 participants and an odds ratio (OR) of 4.5 based on a previous study [22].

### 2.2 Variables

#### 2.2.1 Dependent variable: Mortality

Mortality was defined as the death of older adults by all causes registered by the Epidemiology Office of CEMENA during the study period.

#### 2.2.2 Exposure variables

##### 2.2.2.1 Frailty

Exhaustion: this was defined using three questions from the Center for Epidemiological Studies depression scale (CES-D) [25]. These included: a) Do you feel full of energy? b) Do you feel that you cannot move on? c) Do you feel that everything you do is with effort? A positive answer to two or more questions was considered as exhaustion [1]. Weight Loss: assessed by the question “‘Have you recently lost weight such that your clothing has become looser?” A positive response defined the presence of weight loss [26]. Weakness: this was defined as grip strength less than 27 kg in male participants and less than 16 kg in women [27]. Sedentary behavior was defined using the Physical Activity Scale for the Elderly (PASE) as a score of less than 64 in men and less than 52 in women [28, 29]. Slow gait speed: this was evaluated as gait speed in four meters. Slow gait speed was considered as a speed less than 0.8 m/s or the impossibility to complete the test [27]. The highest time recorded in each participant was registered. Participants were considered frail when three or more criteria were present.

##### 2.2.2.2 Depression

We employed the Yesavage ultra-short scale, which consists of five questions. A score of three or more was considered positive for depressive symptoms [30].

##### 2.2.2.3 Depressed frail phenotype

The depressed frail phenotype was defined as the presence of both depression and frailty in the participants [22].

#### 2.2.3 Other covariates

##### 2.2.3.1 Sociodemographic characteristics

The following sociodemographic characteristics were collected: gender (male, female), age (60–70 years, 71–80 and ≥81), marital status (single, married/cohabitating, divorced/widower), educational level (≤11 years or >11), living alone (no, yes).

##### 2.2.3.2 Medical and personal history

We collected the following comorbidities from the medical records: type 2 diabetes mellitus (T2DM), chronic kidney disease, arterial insufficiency, heart failure, high blood pressure (HBP), chronic obstructive pulmonary disease (COPD), urinary incontinence, knee osteoarthritis, and overweight/obesity. Then, we created a variable that grouped the comorbidities in 0, 1, 2 and ≥3.

Likewise, a history of depression, tobacco consumption (no, yes), alcohol consumption (no, yes), hospitalizations in the last year (no, yes), falls in the last year (no, yes) and the number of medications prescribed were included.

##### 2.2.3.3 Functional assessment

Social support was assessed using the Edmonton frailty scale (no, yes) [28]. Disability to basic activities of daily living (BADL) was measured using the Barthel index, and participants with a score less than 100 were considered dependent [31]. In addition, cognitive impairment was assessed using the Montreal Cognitive Assessment (MoCA) [32].

### 2.3 Statistical analysis

We used the statistical package STATA v.14 for the analyses. The descriptive results of the qualitative variables were presented using relative and absolute frequencies, while the quantitative variables were described using measures of central tendency and dispersion. Pearson's chi-square test was applied to compare the characteristics of the participants according to the exposure variables (frailty, depression, and depressed frail phenotype) and mortality. Likewise, differences between the numerical variables and the exposure and outcome variables were evaluated using the student's t test or the Mann Whitney U test, according to the normal distribution of the numerical variable. Twelve Cox regression models (six crude and six adjusted) were carried out to evaluate the association between the depressed frail phenotype (and its components), with the incidence of mortality in the participants. The adjusted model included the following variables: sex, age, living alone, comorbidities, number of drugs prescribed, and falls in the last year. These variables were chosen according to their association described in the literature [22]. The association measure reported was the hazard ratio (HR) with its respective 95% confidence interval (95%CI). Kaplan-Meier curves graphed the participants' survival according to the presence of the depressed frail phenotype, and they were compared using the Log-rank test.

### 2.4 Ethical aspects

This study was approved by the ethics committee of the *Universidad Científica del Sur* (356-2020-PRE15) located in Lima, Peru. Since we analyzed a secondary database, we did not collect new information. Before entering the study, the participants provided signed informed consent.

## 3 Results

### 3.1 General characteristics of the sample and bivariate analysis according to the exposure variables

We included 946 older adults in the analysis with an average follow-up of 2.2 years. The mean age was 78.0 ± 8.5 and 559 (59.1%) were male. Likewise, 757 (80.0%) were married, 154 (16.3%) lived alone, 266 (28.1%) had three or more comorbidities, and 203 (21.5%) had a history of depression. Furthermore, 582 (61.5%) had functional dependence in BADL and only 90 (9.5%) did not have social support. Regarding the frailty components, 209 (22.1%) had exhaustion, 299 (31.6%) reported weight loss, and 274 (29.0%) had weakness, while 408 (43.1%) were sedentary and 101 (10.7%) had a slow gait speed. In total, 148 (15.6%) were found to be frail, 231 (24.4%) had depressive symptoms, 105 (11.1%) had the depressed frail phenotype and 79 (8.3%) participants died during follow-up (Table 1). The bivariate analysis between covariates and exposure variables is shown in Table 2*.*

**Table 1:** Descriptive analysis of the study variables (n = 946).

Variables	n	%
Sex		
Female	387	40.9
Male	559	59.1
Age	78.0 ± 8.5^4^	
60–70 years old	159	16.8
71–80 years old	448	47.4
≥81 years old	339	35.8
Marital status		
Single	31	3.3
Married/Cohabitating	757	80.0
Divorced/Widower	158	16.7
Educational level		
≤11 years	197	20.8
>11 years	749	79.2
Living alone		
No	792	83.7
Yes	154	16.3
Comorbidities	2 (1–3)^5^	
0	77	8.1
1	309	32.7
2	294	31.1
≥3	266	28.1
History of depression		
No	743	78.5
Yes	203	21.5
BMI^1^	26.2 ± 5.4^4^	
History of tobacco consumption		
No	258	27.3
Yes	688	72.7
History of alcohol consumption		
No	523	55.3
Yes	423	44.7
Functional dependance in BADL^2^		
No	364	38.5
Yes	582	61.5
Hospitalizations in the last year		
No	450	47.6
Yes	496	52.4
Number of drugs prescribed	3 (2–5)^5^	
Social support		
No	90	9.5
Yes	856	90.5
MoCA^3^	27 (23–28)^5^	
Exhaustion		
No	737	77.9
Yes	209	22.1
Weight loss		
No	647	68.4
Yes	299	31.6
Weakness		
No	672	71.0
Yes	274	29.0
Sedentary behavior		
No	538	56.9
Yes	408	43.1
Slow gait speed		
No	845	89.3
Yes	101	10.7
Falls in the last year		
No	358	37.8
Yes	588	62.2
Mortality		
No	867	91.7
Yes	79	8.3

1 Body mass index.

2 Basic activities of daily life.

3 Montreal Cognitive Assessment.

4 Mean ± standard deviation.

5 Median (p25–p75).

**Table 2:** Descriptive and bivariate analyses based on the exposure variables (n = 946).

Variables	Depressed frail phenotype		P value	Frailty		P value	Depressive symptoms		P value
	No 88.9% (n = 841)	Yes 11.1% (n = 105)		No 84.4% (n = 798)	Yes 15.6% (n = 148)		No 75.6% (n = 715)	Yes 24.4% (n = 231)	
Sex			0.002			<0.001			0.700
Female	359 (42.7)	28 (26.7)		347 (43.5)	40 (27.0)		290 (40.6)	97 (42.0)	
Male	482 (57.3)	77 (73.3)		451 (56.5)	108 (73.0)		425 (59.4)	134 (58.0)	
Age	77.6 ± 8.4^4^	81.1 ± 8.5^4^	<0.001	77.5 ± 8.4^4^	80.6 ± 8.5^4^	<0.001	77.6 ± 8.5^4^	79.3 ± 8.3^4^	0.008
60–70 years old	151 (18.0)	8 (7.6)	0.001	147 (18.4)	12 (8.1)	<0.001	132 (18.5)	27 (11.7)	0.034
71–80 years old	405 (48.2)	43 (41.0)		384 (48.1)	64 (43.2)		338 (47.3)	110 (47.6)	
≥81 years old	285 (33.9)	54 (51.4)		267 (33.5)	72 (48.6)		245 (34.2)	94 (40.7)	
Marital status			0.094			0.649			0.458
Single	29 (3.4)	2 (1.9)		28 (3.5)	3 (2.0)		26 (3.7)	5 (2.2)	
Married/Cohabiting	679 (80.7)	78 (74.3)		637 (79.8)	120 (81.1)		573 (80.1)	184 (79.6)	
Divorced/Widower	133 (15.8)	25 (23.8)		133 (16.7)	25 (16.9)		116 (16.2)	42 (18.2)	
Educational level			0.191			<0.001			0.724
≤11 years	170 (20.2)	27 (25.7)		147 (18.4)	50 (33.8)		147 (20.6)	50 (21.6)	
>11 years	671 (79.8)	78 (74.3)		651 (81.6)	98 (66.2)		568 (79.4)	181 (78.4)	
Living alone			0.047			0.086			0.623
No	697 (82.9)	95 (90.5)		661 (82.8)	131 (88.5)		601 (84.1)	191 (82.7)	
Yes	144 (17.1)	10 (9.5)		137 (17.2)	17 (11.5)		114 (15.9)	40 (17.3)	
Comorbidities	2 (1–3)^5^	2 (1–3)^5^	0.579	2 (1–3)^5^	2 (2–3)^5^	0.727	2 (1–3)^5^	2 (1–3)^5^	0.358
0	69 (8.2)	8 (7.6)	0.997	66 (8.3)	11 (7.4)	0.977	57 (8.0)	20 (8.6)	0.620
1	275 (32.7)	34 (32.4)		259 (32.4)	50 (33.8)		239 (33.4)	70 (30.3)	
2	261 (31.0)	33 (31.4)		249 (31.2)	45 (30.4)		225 (31.5)	69 (29.9)	
≥3	236 (28.1)	30 (28.6)		224 (28.1)	42 (28.4)		194 (27.1)	72 (31.2)	
History of depression			0.031			0.033			0.510
No	652 (77.5)	91 (86.7)		617 (77.3)	126 (85.1)		558 (78.0)	185 (80.1)	
Yes	189 (22.5)	14 (13.3)		181 (22.7)	22 (14.9)		157 (22.0)	46 (19.9)	
BMI^1^	26.2 ± 5.3^4^	26.5 ± 5.9^4^	0.501	26.2 ± 5.3^4^	26.3 ± 5.8^4^	0.777	26.2 ± 5.1^4^	26.3 ± 6.1^4^	0.780
History of tobacco consumption			<0.001			<0.001			0.497
No	212 (25.2)	46 (43.8)		183 (22.9)	75 (50.7)		191 (26.7)	67 (29.0)	
Yes	629 (74.8)	59 (56.2)		615 (77.1)	73 (49.3)		524 (73.3)	164 (71.0)	
History of alcohol consumption			0.526			0.021			0.338
No	468 (55.6)	55 (52.4)		454 (56.9)	69 (46.6)		389 (54.4)	134 (58.0)	
Yes	373 (44.4)	50 (47.6)		344 (43.1)	79 (53.4)		326 (45.6)	97 (42.0)	
Functional dependence in BADL^2^			0.580			0.574			0.059
No	321 (38.2)	43 (41.0)		304 (38.1)	60 (40.5)		263 (36.8)	101 (43.7)	
Si	520 (61.8)	62 (59.0)		494 (61.9)	88 (59.5)		452 (63.2)	130 (56.3)	
Hospitalizations in the last year			0.844			0.519			0.662
No	401 (47.7)	49 (46.7)		376 (47.1)	74 (50.0)		343 (48.0)	107 (46.3)	
Yes	440 (52.3)	56 (53.3)		422 (52.9)	74 (50.0)		372 (52.0)	124 (53.7)	
Number of drugs prescribed	3 (2–4)^5^	7 (6–8)^5^	<0.001	3 (2–4)^5^	7 (5–8)^5^	<0.001	3 (2–4)^5^	7 (5–8)^5^	<0.001
Social support			<0.001			<0.001			0.435
No	69 (8.2)	21 (20.0)		51 (6.4)	39 (26.4)		65 (9.1)	25 (10.8)	
Yes	772 (91.8)	84 (80.0)		747 (93.6)	109 (73.6)		650 (90.9)	206 (89.2)	
MoCA^3^	27 (24–29)^5^	19 (17–20)^5^	<0.001	27 (25–29)^5^	20 (19–22) 5	<0.001	27 (25–29)^5^	20 (19–22)^5^	<0.001
Falls in the last year			0.787			0.713			0.825
No	317 (37.7)	41 (39.0)		300 (37.6)	58 (39.2)		272 (38.0)	86 (37.2)	
Yes	524 (62.3)	64 (61.0)		498 (62.4)	90 (60.8)		443 (62.0)	145 (62.8)	
Mortality			<0.001			<0.001			<0.001
No	804 (95.6)	63 (60.0)		761 (95.4)	106 (71.6)		715 (100)	152 (65.8)	
Yes	37 (4.4)	42 (40.0)		37 (4.6)	41 (27.7)		0 (0)	79 (34.2)	

1 Body mass index.

2 Basic activities of daily life.

3 Montreal Cognitive Assessment.

4 Mean ± standard deviation.

5 Median (p25–p75).

### 3.2 Bivariate analysis according to mortality in the study sample

Mortality was found to be higher in patients with the depressed frail phenotype (53.2% vs. 46.8%; p < 0.001) compared to those who did not have this conditions. In addition, the incidence of mortality was higher in older adults with exhaustion (51.9% vs. 48.1%; p < 0.001), weight loss (43.0% vs. 57.0%; p = 0.022), weakness (62.0% vs. 26.0%; p < 0.001), sedentary behavior (78.5% vs. 39.9%; p < 0.001) and slow gait speed (11.4% vs. 10.6%; p = 0.830) compared to those who did not have these geriatric syndromes (Table 3).

**Table 3:** Descriptive and bivariate analyses of the study variables based on all-cause mortality (n = 946).

Variables	Mortality		P value
	No 91.7% (n = 867)	Yes 8.3% (n = 79)	
Depressed frail syndrome			<0.001
No	804 (92.7)	37 (46.8)	
Yes	63 (7.3)	42 (53.2)	
Frailty			<0.001
No	761 (87.8)	37 (46.8)	
Yes	106 (12.2)	42 (53.2)	
Depressive symptoms			<0.001
No	715 (82.5)	0 (0)	
Yes	152 (17.5)	79 (100)	
Sex			0.204
Female	360 (41.5)	27 (34.2)	
Male	507 (58.5)	52 (65.8)	
Age	77.8 ± 8.5 4	80.4 ± 7.8 4	0.008
60–70 years old	154 (17.8)	5 (6.3)	0.024
71–80 years old	409 (47.2)	39 (49.4)	
≥81 years old	304 (35.1)	35 (44.3)	
Marital status			0.260
Single	28 (3.2)	3 (3.8)	
Married/Cohabiting	689 (79.5)	68 (86.1)	
Divorced/Widower	150 (17.3)	8 (10.1)	
Educational level			0.062
≤11 years	187 (21.6)	10 (12.7)	
>11 years	680 (78.4)	69 (87.3)	
Living alone			0.023
No	733 (84.5)	59 (74.7)	
Yes	134 (15.5)	20 (25.3)	
Comorbidities	2 (1–3) 5	2 (1–2) 5	0.323
0	71 (8.2)	6 (7.6)	0.390
1	281 (32.4)	28 (35.4)	
2	265 (30.6)	29 (36.7)	
≥3	250 (28.8)	16 (20.3)	
History of depression			0.989
No	681 (78.6)	62 (78.5)	
Yes	186 (21.4)	17 (21.5)	
BMI^1^	26.2 ± 5.2 4	26.1 ± 6.9 4	0.846
History of tobacco consumption			0.046
No	244 (28.1)	14 (17.7)	
Yes	623 (71.9)	65 (82.3)	
History of alcohol consumption			0.432
No	476 (54.9)	47 (59.5)	
Yes	391 (45.1)	32 (40.5)	
Functional dependency in BADL^2^			<0.001
No	308 (35.5)	56 (70.9)	
Yes	559 (64.5)	23 (29.1)	
Hospitalizations in the last year			0.544
No	415 (47.9)	35 (44.3)	
Yes	452 (52.1)	44 (55.7)	
Number of prescribed drugs	3 (2–4) 5	8 (7–8) 5	<0.001
Social support			0.159
No	86 (9.9)	4 (5.1)	
Yes	781 (90.1)	75 (94.9)	
MoCA^3^	27 (24–28) 5	20 (18–20) 5	<0.001
Exhaustion			<0.001
No	699 (80.6)	38 (48.1)	
Yes	168 (19.4)	41 (51.9)	
Weight loss			0.022
No	602 (69.4)	45 (57.0)	
Yes	265 (30.6)	34 (43.0)	
Weakness			<0.001
No	642 (74.0)	30 (38.0)	
Yes	225 (26.0)	49 (62.0)	
Sedentary behavior			<0.001
No	521 (60.1)	17 (21.5)	
Yes	346 (39.9)	62 (78.5)	
Slow gait speed			0.830
No	775 (89.4)	70 (88.6)	
Yes	92 (10.6)	9 (11.4)	
Falls in the last year			0.153
No	334 (38.5)	24 (30.4)	
Yes	533 (61.5)	55 (69.6)	

1 Body mass index.

2 Basic activities of daily life.

3 Montreal Cognitive Assessment.

4 Mean ± standard deviation.

5 Median (p25–p75).

### 3.3 Depressed frail phenotype as a predictor of mortality in older adults

**Figure 1:**
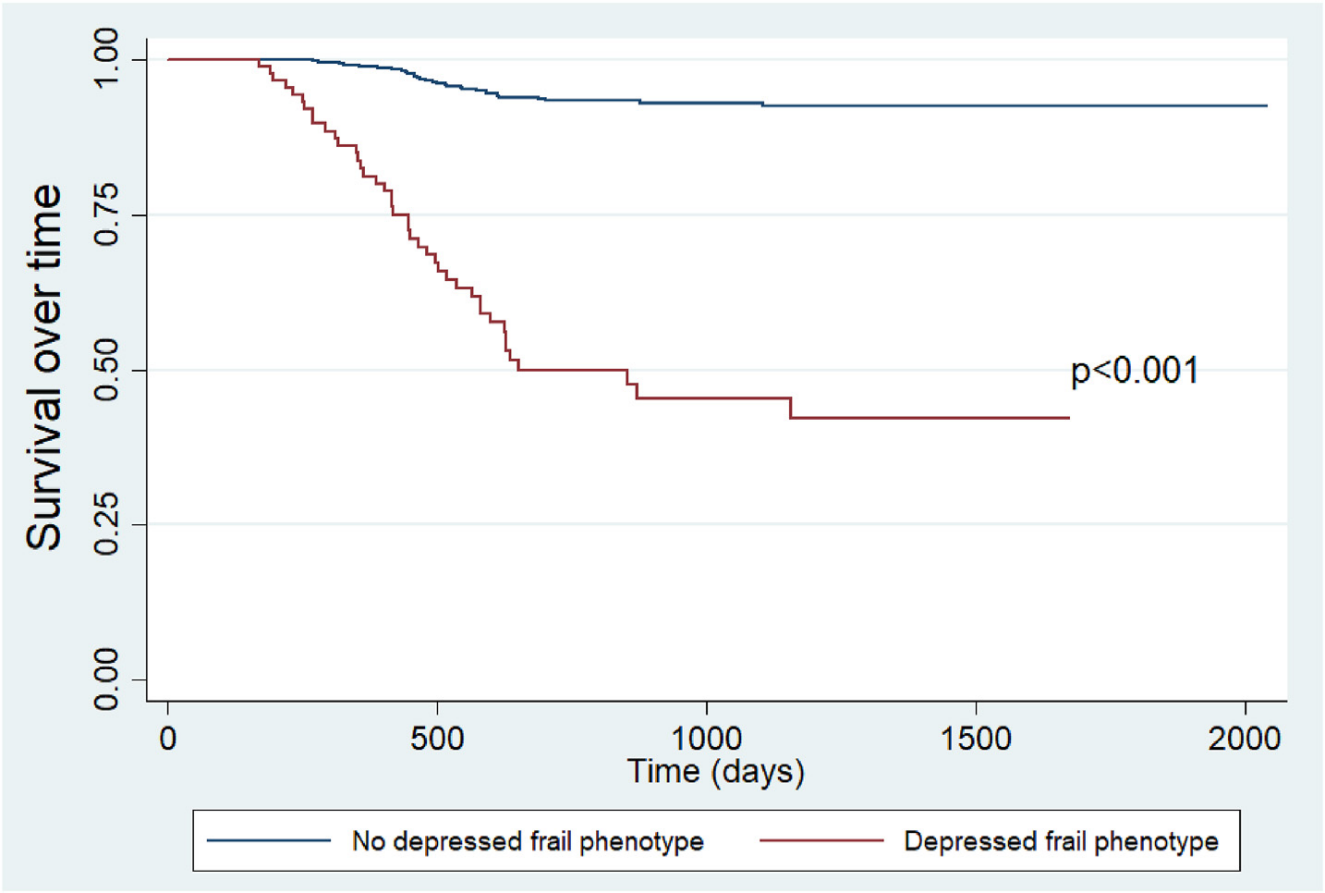
Kaplan-Meier survival curves according to the depressed frail phenotype.

In the adjusted Cox regression analysis, the depressed frail phenotype (aHR = 3.53; 95%CI: 2.07–6.00; p < 0.001) was found to be a predictor of mortality in older adults. In relation to the frailty components, we found that participants with exhaustion and depression (aHR = 1.99; 95%CI: 1.17–3.40; p = 0.011), weight loss and depression (aHR = 2.10; 95%CI: 1.24–3.55; p = 0.006), weakness and depression (aHR = 4.11; 95%CI: 2.31–7.34; p < 0.001), sedentary behavior and depression (aHR = 6.00; 95%CI: 3.33–10.84; p < 0.001) and slow gait speed and depression (aHR = 3.12; 95%CI: 1.54–6.30; p = 0.002) (Table 4) had a higher risk of death. Likewise, we found a greater risk of mortality in the depressed frail phenotype group according to the Kaplan-Meier curve (p < 0.001) (Figure 1).

**Table 4:** Cox regression models to evaluate the association between the depressed frail phenotype and the risk of mortality in the study sample.

	Crude			Adjusted		
**Depressed frail phenotype**	cHR	95%CI	P value	aHR^1^	95%CI	P value
No	Reference	–	–	Reference	–	–
Yes	10.88	6.99–16.94	<0.001	3.53	2.07–6.00	<0.001
**Depressed frail phenotype components**						
**Exhaustion + depression**						
No	Reference	–	–	Reference	–	–
Yes	9.38	6.03–14.61	<0.001	1.99	1.17–3.40	0.011
**Weight loss + depression**						
No	Reference	–	–	Reference	–	–
Yes	8.51	5.44–13.30	<0.001	2.10	1.24–3.55	0.006
**Weakness + depression**						
No	Reference	–	–	Reference	–	–
Yes	11.90	7.54–18.76	<0.001	4.11	2.31–7.34	<0.001
**Sedentary behavior + depression**						
No	Reference	–	–	Reference	–	–
Yes	19.60	11.43–33.62	<0.001	6.00	3.33–10.84	<0.001
**Slow gait speed + depression**						
No	Reference	–	–	Reference	–	–
Yes	4.46	2.23–8.94	<0.001	3.12	1.54–6.30	0.002

HR: hazard ratio; aHR: adjusted hazard ratio; CI: confidence interval.

1 Adjusted for: sex, age, living alone, comorbidities, number of prescribed drugs and falls in the last year.

## 4 Discussion

### 4.1 Main findings

To our knowledge, this is the first study evaluating this association in older Latin American adults, for which we consider our findings to be novel and of great utility for the identification of vulnerable groups at higher risk of mortality. Thus, it would be relevant to prioritize screening for depression and frailty, as well as the coexistence of both geriatric syndromes in comprehensive geriatric evaluations.

### 4.2 Comparison with previous studies

In this study we found that the prevalence of depression, frailty, and the depressed frail phenotype was 24.4%, 15.6%, and 11.1%, respectively. The prevalence of frailty and depression is lower than that reported in previous studies carried out in Spain and Denmark [15, 22]. However, it is higher than what was reported in an Australian study [20]. It should be noted that no previous study has reported the prevalence of depressed frail phenotype. Likewise, a systematic review found a prevalence of frailty in older South American adults of 21.7%, being higher than that reported in our study [6]. On the other hand, it was lower than the prevalence of frailty of 13% found in a systematic review using the Fried phenotype [5].

We found an association between the depressed frail phenotype and mortality in older adults in Peru. This finding to similar to the results of previous studies carried out in Australia [20], Denmark [15] and Spain [22]. However, only two of these studies estimated an association measure [15, 22], and all three were carried out in high-income countries. Only one study evaluated the impact of depression on each frailty component [15], and the follow-up time in this study was shorter compared to the previous studies [15, 20, 22]. It should be noted that we employed the Fried phenotype to evaluate frailty, while in previous studies this variable was measured with instruments based on deficit accumulation, which are not the most widely accepted [33, 34]. We evaluated the risk of mortality according to each component of the depressed frail phenotype, determining that sedentary behavior, weakness and slow gait speed were the components with the highest risk, which differs from a previous study [15]. To our knowledge, this is the first study to evaluate the impact of depressed frail phenotype on the mortality of older adults in Latin America, providing novel data on the impact of this condition on the population of our region.

We found that older adults with depressed frail phenotype and who died were older, had a greater number of prescribed drugs, and a higher frequency of disability. Previous studies are consistent with our findings, where increasing age was associated with a higher risk of frailty and mortality [35]. Likewise, a study carried out in Spanish older adults found that disability, and polypharmacy were risk factors for mortality, independently of depressed frail phenotype [22]. In addition, depression has been described as a risk factor for disability and mortality in older adults [36], therefore it is necessary to prevent it.

### 4.3 Interpretation of results

The depressed frail phenotype is a condition that generates a high risk of mortality in older adults. Frailty is characterized by a deficit of homeostasis in relation to an adverse event in older adults, which, added to depression, can increase the risk of mortality [13, 14, 37]. The pathophysiology of the depressed frail phenotype as a predictor of mortality is based on the chronic inflammatory state that is usually present in older adults and is associated with frailty and depression. This inflammatory state affects the dopaminergic system and the basal ganglia of older adults, which together with comorbidities such as type 2 diabetes mellitus, metabolic syndrome and low level of physical activity, increases the risk of adverse events. Other theories to explain the development of this syndrome are mitochondrial and dopaminergic dysfunction, hormonal dysregulation of the hypothalamic pituitary adrenal axis, and accelerated cellular aging [14]. Similarly, vascular involvement can produce depressive symptoms and alterations in physical and cognitive performance [38, 39].

A potential cycle of the depressed frail phenotype has also been described, in which depression unidirectionally causes weight loss and a reduction in physical activity, generating sarcopenia, decreased metabolic rate and strength, and greater exhaustion in older adults due to a deficiency in adenosine triphosphate production [14, 40, 41]. Taking this into account, the probability of falls increases, with the subsequent loss of immobilization. Eventually body strength decreases, creating a cycle. Immobilized older adult suffer from slow gait speed and perform less physical activity, leading to depression, thus forming a vicious circle [1]. This is consistent with our findings, in which the most important components of the depressed frail phenotype were sedentary behavior, weakness and slow gait speed.

We must mention that both syndromes are independent of each other, but they can simultaneously coexist in older adults. Another possibility is that one acts as a cause for the other, which is why it is relevant to carry out more longitudinal studies to verify this hypothesis and determine triggering conditions. Finally, depression and frailty can manifest in different ways, when in fact they are part of the same disorder.

### 4.4 Relevance in clinical practice

The increase in the prevalence of frailty, depression, and the depressed frail phenotype indicates the relevant need to consider certain aspects of clinical practice and the management of older adults. While treatment with antidepressants has shown to improve the management of depression [42], this therapy may not be applicable in frail people, who have a higher risk of adverse outcomes [43]. On the other hand, nutritional interventions and exercise aimed at improving muscle strength in the lower extremities, gait speed and preventing future falls, have shown good results [44, 45]. Nonetheless, the depressed population is not usually included in these programs [46, 47, 48], even though they would provide a greater impact to combat sedentary behavior and exhaustion. There are still gaps in relation to the usefulness of pharmacological and non-pharmacological interventions to improve the quality of life of older adults with the depressive frail phenotype, as well as to avoid a greater impact of depression in frail individuals, and vice versa. Nonetheless, the use of antidepressants could have serious adverse effects in frail and comorbid populations, such as an increased risk of hyponatremia, bleeding, vision disturbances, memory loss, delirium, and falls [49, 50]. This situation highlights the need for more intervention studies including patients with the depressed frail phenotype due to the high risk of mortality.

Various non-pharmacological interventions are available for the prevention and treatment of depression in older adults, such as exercise, cognitive behavioral therapy (CBT), behavioral activation, problem-solving therapy, and bright-light therapy [51]. The positive effect of CBT on depressive symptoms in older adults has been described in previous systematic reviews and meta-analyses [51, 52, 53, 54, 55]. However, more studies are needed to evaluate the safety and efficacy of these interventions even in older adults with depressed frail phenotype.

### 4.5 Strengths and limitations

Our study has some limitations: 1) The population consisted of retired marines and their families, who share certain characteristics that may vary compared to the general population of older adults in Peru and may therefore not be representative. 2) We analyzed a secondary database, in which relevant variables for this study could not be measured or included, such as the use of antidepressants and other comorbidities. 3) Depression was not evaluated using the criteria of the Diagnostic and Statistical Manual of Mental Disorders, Fifth Edition; however, we employed a screening scale with adequate diagnostic performance which has been widely used in previous studies [22,50]; 4) We did not perform annual measurements to record the appearance of new comorbidities, the occurrence of falls and hospitalizations, which could be relevant for the incidence of mortality; 5) The follow-up time to assess mortality may not be sufficient in certain cohort groups. Despite the limitations described, this is the first study in Latin America to evaluate the depressed frail phenotype as a predictor of mortality in older adults and our results provide relevant information to understand this phenomenon.

## 5 Conclusions

We found that the depressed frail phenotype and its components were risk factors for mortality in older adults. Sedentary behavior, weakness, and slow gait speed had the greatest impact on mortality, for which interventions in these components should be prioritized. It is necessary to develop more longitudinal studies that allow estimating the impact of this syndrome on mortality and evaluate interventions to improve quality of life and reduce the risk of adverse outcomes.

## Declarations

### Author contribution statement

Gabriel A.J. Vasquez-Goñi, Basilio M. Papuico-Romero: Conceived and designed the analysis; Wrote the paper.

Diego Urrunaga-Pastor: Conceived and designed the analysis; Analyzed and interpreted the data; Wrote the paper.

Fernando M. Runzer-Colmenares: Conceived and designed the analysis; Analyzed and interpreted the data; Contributed analysis tools or data; Wrote the paper.

José F. Parodi: Analyzed and interpreted the data; Contributed analysis tools or data; Wrote the paper.

### Funding statement

This research did not receive any specific grant from funding agencies in the public, commercial, or not-for-profit sectors.

### Data availability statement

The authors do not have permission to share data.

### Declaration of interests statement

The authors declare no conflict of interest.

### Additional information

No additional information is available for this paper.
